# Improvement in OCD symptoms associated with serotoninergic psychedelics: a retrospective online survey

**DOI:** 10.1038/s41598-023-39812-0

**Published:** 2023-08-17

**Authors:** Anne Buot, Cecile Pallares, Alina Oganesyan, Charles Dauré, Valérie Bonnelle, Eric Burguière, Joao Flores Alves Dos Santos, Karim N’Diaye, Michael Ljuslin, Pauline Smith, Vincent Verroust, Benjamin Wyplosz, Margot Morgiève, Luc Mallet

**Affiliations:** 1 Sorbonne Université, Institut du Cerveau - Paris Brain Institute - ICM, Inserm, CNRS, Paris, France; 2 Université Paris Cité, CNRS, Inserm, Cermes3, Paris, France; 3 Beckley Foundation, Oxford, UK; 4 Department of Mental Health and Psychiatry, University of Geneva, Geneva, Switzerland; 5 Service de Médecine Palliative, Département de Réadaptation Et Gériatrie, Hôpitaux Universitaires de Genève, Geneva, Switzerland; 6 Environmental Justice Program, Georgetown University, Washington, D.C USA; 7 RESPADD, Paris, France; 8 UR PSYCOMADD- CHU Paul Brousse, Villejuif, France; 9 Université de Picardie-Jules Vernes, Amiens, France; 10 Hôpital Bicêtre, Assistance Publique-Hôpitaux de Paris, Le Kremlin-Bicêtre, France; 11 Univ Paris-Est Créteil, DMU IMPACT, Département Médical-Universitaire de Psychiatrie Et d’Addictologie, Hôpitaux Universitaires Henri Mondor - Albert Chenevier, Assistance Publique-Hôpitaux de Paris, Créteil, France

**Keywords:** Obsessive compulsive disorder, Therapeutics

## Abstract

A renewed interest in the use of psychedelics for treating obsessive compulsive disorder (OCD) has emerged in the last 20 years. But pre-clinical and clinical evidence remain scarce, and little is known about the factor determining the magnitude and persistence of the therapeutic effect. We therefore designed a retrospective online survey to explore, in the general population using psychoactive drugs, their impact on OCD symptoms. We also assessed the attitude of the participants towards the substance in term of frequency of intakes. In a sample of 174 participants, classic psychedelics were reported as the only substances effective at reducing OCD symptoms. In classic psychedelics users, symptoms reduction was associated with the intensity of acute effects, itself correlated to the dose. Reports on the persistence of the therapeutic effect varied from weeks to months, but we could not find any predicting factor. Finally, the occurrence and frequency of subsequent intakes, which seemed to be limited in our sample, were predicted by the magnitude and persistence of the therapeutic effect, respectively. Our observations support the hypothesis of classic psychedelics efficacy in reducing OCD symptoms but a careful evaluation of the persistence of this effect is still needed.

## Introduction

Obsessive–compulsive disorder (OCD) is a debilitating psychiatric condition, characterized by intrusive thoughts called obsessions, repetitive behaviors called compulsions, and avoidance behaviors^1^. The main therapeutical approach for OCD relies on psychological interventions, mainly cognitive and behavioral therapy (CBT), and pharmacological interventions, with first-line use of selective serotonin reuptake inhibitors (SSRIs) given at higher doses than in other indications^2,3^. OCD tends to be chronic and persistent, and impairment is common even with the best medical therapy^3^. Disadvantages of current medications include difficulty in finding an effective medication and long delays of several weeks or even months before symptoms’ improvement^4^. Finally, 30–40% of patients do not respond at all to SSRI treatment and many of the patients who do respond continue to have problematic residual symptoms^3^. OCD is therefore one of the few psychiatric disorders for which invasive brain surgery is an accepted treatment option, despite difficulties of access to this treatment as part of research protocols^5^. For these reasons, new treatment strategies that could act both on pharmacological and psychotherapeutic dimensions with a good level of acceptance are of great interest.

In this context, it has been suggested that classic psychedelics, such as lysergic acid diethylamide (LSD) and psilocybin (the main active compound of so-called magic mushrooms), might be used to decrease OCD symptoms. Classic psychedelics are also called serotoninergic hallucinogens since these substances are thought to exert their effects by a partial agonist action on 5-hydroxytryptamine (5-HT) 2A receptors. Some evidence of the therapeutic property of classic psychedelics came from clinical studies from the 70’s^6–8^ but also from more recent case reports^9–11^ and an online survey^12^. Evidence remains scarce with the most substantial study being an open-label trial, in which 9 patients with moderate to severe OCD were exposed to various doses of psilocybin ranging from low sub-hallucinogenic to high hallucinogenic ones^13^. This trial suggested that psilocybin might be efficient in decreasing OCD symptoms, but the follow-up was limited to 24 h and long-term effects were not assessed. Also placebo or expectation effects, which are important confounding factors in psychedelic assisted psychotherapy^14^, were not assessed. But controlled studies are underway with multiple ongoing clinical trials^15–17^. Evidence from preclinical studies is also scarce, with some studies assessing the effect of psychedelics in animal models of compulsive behavior such as the serotonin transporter heterozygous (Sert^+^/^−^) mice^18^ or on the marble burying test^19,20^. Whereas serotoninergic psychedelics such as DOI (2,5-dimethoxy-4-iodoamphetamine) and psilocybin seemed to be efficient in decreasing digging behavior, LSD had no effect on grooming behavior in the Sert heterozygous model.

Since psychedelics have been classified under Schedule I in the 1971 conventions of the United Nations^21^ and remain illegal in many countries, observational studies such as anonymous online surveys have been used to assess the use, misuse and potential therapeutic properties of psychedelics^22–26^. The present retrospective, online and anonymous survey aimed to evaluate, in the general population with OCD symptoms, which substances among multiple psychoactive drugs might have a therapeutic effect. The second aim was to explore whether these putative improvements were related to specific characteristics of the acute subjective experience, such as the intensity and pleasantness of acute effects, as well as the mindset (i.e., the expectations or state of mind when taking the substance) and setting (i.e., the context of the intake). The latter two parameters were explored since it has been considered as important factors in shaping the acute response to psychedelics^27^. The third aim was to evaluate the occurrence and frequency of substance intakes.

## Methods

### Survey administration

The survey was designed by the authors with a French and an English version. Our research procedure was in accordance with the relevant guidelines and regulations (1964 Declaration of Helsinki and its later amendments, methodological reference of CNIL for data management), and was approved by the Research Ethics Committee of Sorbonne University (https://www.sorbonne-universite.fr/en/universite/our-commitments/research-ethics-committee, CER n°2021–067). Specifically, all data was confidential—no IP address or other identifying data was acquired. Eligible participants had to be more than 18 years old, have OCD symptoms and at least one experience with one of the following psychoactive drugs: LSD, ecstasy, MDMA, psilocybin, DMT, ayahuasca, ketamine, 2C-B, Salvia divinorum, mescaline, ibogaine.

From September 2021 to March 2022, the survey was advertised online on several platforms including webpages dedicated to OCD such as the AFTOC (www.aftoc.org), AETOC (www.aetoc.ch), FQTOC (www.fqtoc.com), LIGUETOC (www.liguetoc.wordpress.com) which are the French, Swiss, Canadian and Belgian association for people with OCD, respectively; webpages dedicated to usage and research on psychoactive drugs such as of the Beckley Foundation (www.beckleyfoundation.org), the French psychedelic society (www.societepsychedelique.fr), the Swiss psychedelic society (www.eleusis-society.ch), the French forum for psychoactive drugs users (www.psychonaut.fr). The survey was also posted on Facebook pages dedicated to OCD (French pages: *Lutter contre le TOC*, *Troubles anxieux*, *AFTOC*; English pages: *Obsessive Compulsive Disorder sufferers friendship and support group*, *OCD Only support group, Intrusive Thoughts and Pure OCD, OCD Recovery Group, OCD support group*)*.*

At the beginning of the survey, a short introduction presented the aim of the study (*To characterize the use of psychedelic substances in people with OCD and their possible effects on the disorder*), the inclusion criteria and the general structure of the survey. Links to psychological support websites and phone lines were also provided for participants in need of help regarding OCD or drugs use. To start the survey, participants had to explicitly give their informed consent. The completion of the survey took 15 to 20 min. At any time, participants could interrupt the survey by simply closing their browsers. In this case, data was tagged as incomplete allowing us to detect and discard the participant. Study data were collected and managed using REDCap electronic data capture web application hosted at Paris Brain Institute^28^.

### Measurements

#### Sociodemographic information

In part I, sociodemographic information (i.e., age, gender, height, weight, social status and environment of living) was collected.

#### Health information

In part II, health information was collected. Concerning OCD symptoms, participants were asked if they had been diagnosed, who made the diagnosis (possible choices: *a health professional, yourself, other*) and when. Participants then had to fill in the short version of the Obsessive Compulsive Inventory (OCI-r)^29^ to get a severity score. Participants were also asked whether they were currently in therapy and if yes which type of therapy (possible choices: *cognitive-behavioral therapy (CBT), psychoanalytic-psychodynamic therapy, eye movement desensitization and reprocessing (EMDR), mindfulness (MBCT), systemic or family therapy, relaxation, art therapy, hypnosis, alternative therapies, other*); whether they took medications and if yes which type of medications (possible choices: *antidepressants, neuroleptics, anxiolytics, alternative/complementary medications, other*) and at which frequency (possible choices: *every day, several times a week, more than once a month, once a month, other*).

#### Usage of psychedelics

In part III, the usage of psychoactive drugs was assessed. Participants add to select which substances they had used (multiple answers possible) within the following list: *LSD, ecstasy, 3,4-methylenedioxymethamphetamin (MDMA), psilocybin mushrooms, dimethyltryptamine (DMT), ayahuasca, ketamine, 2CB (4-bromo-2,5-dimethoxyphenetylamine), Salvia divinorum, mescaline, ibogaine, other*.

#### Link between the intake of psychoactive drugs and changes in OCD symptoms

In part IV, the impact of psychoactive drugs on OCD symptoms was assessed. The first question was “*Have you experienced a change in your OCD as a result of taking a psychedelic?*” Participants answering *No* were redirected toward the end of the survey, whereas participants answering *Yes* had to select the substance that induced the strongest changes in OCD symptoms, among the list presented in part III. From there, all subsequent questions/answers were relative to this given substance. Participants who selected a given substance as the most impacting but did not previously select it as a used one were discarded from the analysis for uncoherent responses (n = 4). Changes in OCD symptoms triggered by the intake of the given substance were assessed using 6 items: *negative emotions, obsessions, compulsions/rituals, anxiety, acceptance of condition,* and *avoidance of possible anxiogenic situations.* Each item was scored by moving a cursor on a 100-point scale (2-point step) starting at midpoint (0: *no change*) and ranging from -100 (*Worsening*) to + 100 (*Improvement*). Participants who did not score any of the items were discarded due to missing responses (n = 6). The persistence of these changes was also examined with participants choosing between *a few hours, less than a day, one to three days, three days to a week, between a week and a month, one to three months, more than three months, permanently*.

#### Mindset, setting and dose

The mindset was investigated by asking participants about their expectations before the drug intake (possible choices: *recreational experience, personal development, spiritual or religious purpose, OCD improvement, other therapeutic purpose, curiosity/discovery, none, other*); and the setting by asking participants in which context they had taken the substance (possible choices: *no particular event, at a party or concert, shamanic type ceremony, with therapeutic support, clinical research, retreat, other*). Participants were also asked about the substance dose, the delay between the intake and the appearance of the subjective effects (possible choices: *0–15 min, 15–30 min, 30–60 min, 60–90 min,* > *90 min*), and the duration of the effects (possible choices: *30–60 min, 1–3 h, 3–5 h, 5–12 h,* > *12 h, I don’t know*). Finally, participants were asked if they had taken the substance again and how often by choosing between *no, less than once a year, one to three times a year, once a month, once a week, two to six times a week, daily, other*.

#### Subjective experience

In part V, participants had to rate acute general effects by scoring the 13 following items: *emotional arousal, euphoria/ecstasy/bliss, altered body sensations, changes in vision and/or hearing, musical ecstasy, knowledge/insights on existential subjects (death, love, divine, man's place in the universe), space–time distortion effect, mystical/transcendent/spiritual experience (feeling of unity), trauma resolution, hallucination(s), delirium/confusion, ego dissolution (complete loss of subjective self-identity), bodily boundaries dissolution*. Each item was scored by moving a cursor on a 100-points scale (1-point step) starting from 0 (*Not at all*) to 100 (*Significantly*). Using the same scale, participants also had to rate the acute unpleasant effects on the 21 following items: *Diarrhea, Constipation, Nausea, Vomiting, Headache, Sedation/Drowsiness, Tachycardia, Shaking, Feeling of heat, Feeling of coldness, Difficulty concentrating, Memory impairment, Indigestion/Stomach troubles, Discomfort, Anxiety, Sadness/Melancholy, Revival of traumatic memories, Terror/Paranoia/Panic, Depersonalization, Aggressive behavior towards yourself, Aggressive behavior towards others*.

Additional questions were asked that are not reported here. The full survey is available at the following link: https://osf.io/x2sk7/?view_only=5ef581a60b7a4d5685a2c233a0435a15.

### Data transformation

**Figure 1:**
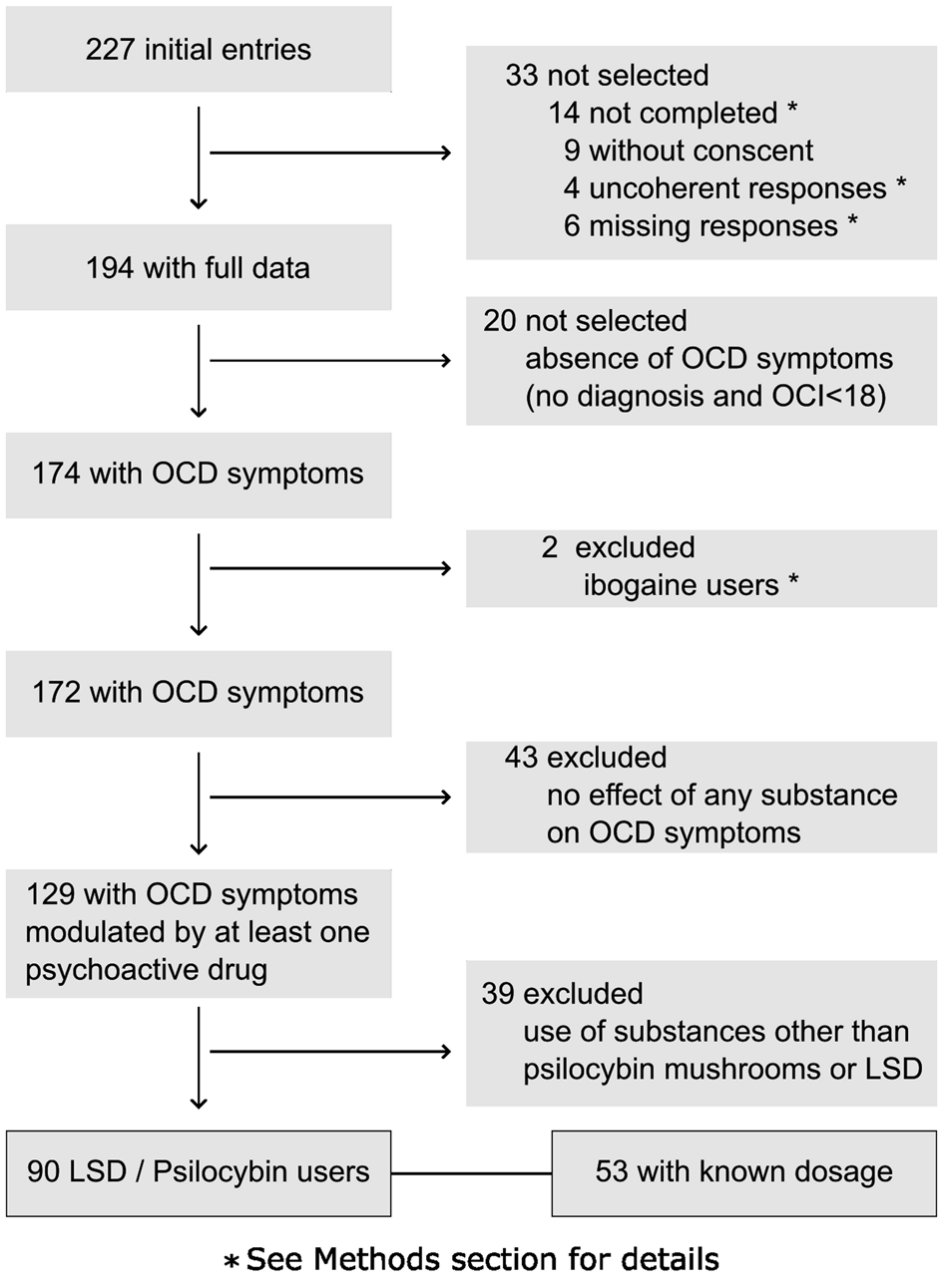
Flow chart showing steps in participants selection.

Data were exported from RedCap and imported in R 3.5.1 (R Core Team 2018). All answers from the French version were translated to match the English one and the two datasets were merged. We checked for duplicates by comparing the combination of age, gender, height and weight across participants. We then screened participants who did not meet the inclusion criteria, did not give their consent, or did not reach the end of the survey (n = 33, Fig. 1). Participants who did not provide answers on some questions but completed the survey until the end were kept for analysis.

When needed, new variables were created to reclassify answers. First, substances were grouped into 5 substance categories based on their chemical properties and subjective effects as follows: *classic psychedelics* including *LSD, psilocybin mushrooms, DMT, mescaline* and *ayahuasca*; *entactogens* including *ecstasy* and *MDMA*; *ketamine*; *delirogens* including *Salvia divinorum* and *Datura*; and *novel psychoactive substances (NPS*) including *2CB.* Participants selecting ibogaine as the most impacting substance were discarded due to the small sample size (n = 2) not being appropriate for regression analyses. Second, to get enough answers per category, persistence data were reclassified into the 4 categories *less than a week, between 1 week and 1 month, between one and three months,* and *more than three months;* and frequency of subsequent intake data were reclassified into the 3 categories *maximum 3 times a year*, *once a month* and *at least once a week.* Third, to get enough answers per category, setting data were reclassified into 3 categories as follow: *No particular event* stayed n*o particular event*, *at a party or concert* became *recreational setting*, and *shamanic type ceremony, with therapeutic support, clinical research* and *retreat* were grouped in the *ceremonial group setting* category. Sixteen answers from participants who selected *other* and provided details could be reclassified in the abovementioned categories based on explanation. Finally, since participants could provide multiple answers when asked about their mindset, we selected the item which was the most relevant for our study: *OCD improvement* and transformed it into a *Yes/No* binary variable. For the magnitude of changes in OCD symptoms, intensity and pleasantness of acute effects, we used the within-participant mean of the ratings on the different subscales: mean of the OCD dimensions’ ratings for the magnitude of changes in OCD, mean of general effects’ ratings for intensity, mean of unpleasant effects’ ratings subtracted from 100 for pleasantness (to get scores that increased with pleasantness).

### Statistical analyses

We then proceeded to statistical analyses. A chi-squared (χ^2^) test was used to test if the proportion of users declaring a given substance as the most impacting one varied according to the substance, and one-sample Student’s t-tests were used to evaluate if the magnitudes of the changes induced by the different substances were significantly different from 0. In subsequent analyses, we focused on LSD and psilocybin mushrooms users (n = 90, Fig. 1). To evaluate which factor might predict the magnitude of the changes in OCD symptoms, a linear regression was used that included 6 independent variables: the substance (*LSD* vs *Psilocybin mushrooms*), the mindset (binary yes/no variable indicating whether subjects expected OCD improvement), the setting (*no particular event, recreational setting, ceremonial group setting*), the intensity and pleasantness of the acute effects and the OCI-r score (all three are continuous variables). A linear regression was also used to evaluate which factor might predict the pleasantness of acute effects, which included 5 independent variables: the substances, the mindset, the setting, the intensity of the acute effects and the OCI-r score. To evaluate which variables might predict the persistence of the changes in OCD symptoms, an ordinal regression was used with 5 regressors: the substances, the intensity and pleasantness of acute effects, the magnitude of changes in OCD symptoms and the OCI-r score. Finally, the occurrence of subsequent intakes and their frequency were also analyzed. The occurrence of subsequent intake was encoded as a binary variable (1 or 0 corresponding to *Yes* or *No*) and was regressed out using a logistic regression with 6 regressors: the substances, the intensity and pleasantness of the acute effects, the OCI-r score, the magnitude and the persistence of the changes in OCD symptoms. Subsequent intake frequency was then analyzed in the subset of participants with subsequent intakes, using an ordinal regression with the 5 following regressors: the substance, the intensity and pleasantness of acute effects, the magnitude and persistence of the changes in OCD symptoms. Since multiple regressions were performed on the same sample of participants (magnitude of the changes in OCD symptoms, pleasantness of acute effects, persistence of the changes in OCD symptoms, occurrence of subsequent intakes), the associated p-values were corrected for multiple comparison using the Benjamini–Hochberg procedure. For these analyses, adjusted p-values are reported. Additionally, multicollinearity between independent variables has been assessed for all regressions using the generalized variance inflation factor (GVIF). All analyses were performed using R and the following packages: *dplyr* for data frame manipulations (v1.0.10), *forcats* for handling categorical variables (v0.5.0) and *ggplot2* for plotting. Basic statistical analyses were performed using the *rstatix* package. Linear and logistic regressions were performed using the *lm* and *glm* functions of the stats package, respectively, and ordered regressions were performed using the *clm* function of the *ordinal* package. GVIF values were computed using the *car* package. To diagnose if all assumptions of linear regressions were met, we inspected the plots representing the relationship between fitted values and residuals, the distribution of the residuals, the homoscedasticity of the residuals and the presence of influential outlying data. For ordinal regressions, we checked the assumption of parallel slopes using the *brant* package.

## Results

### Participants selection

A flow chart representing the selection procedure is shown in Fig. 1. During recruitment, 227 participants entered the survey. Thirty-three participants were discarded because of no consent, incoherent or missing responses, or incomplete questionnaire (see Methods for details). We then selected our population of interest based on OCD symptoms and kept for analyses the data from participants who had either a diagnosis of OCD performed by a health professional (n = 9), or an OCI-r score superior to 18 (n = 62)^29^ or both (n = 103). The rationale was to include participants who had not been screened by a psychiatrist or a psychologist (participants without diagnosis but OCI-r > 18), and participants in remission when filling out the survey (participants with a diagnosis but OCI-r < 18). Using these criteria, we identified 174 participants (76% from the English survey).

### Participants socio-demographic and health characteristics

Participants’ socio-demographic and health information are described in Tables 1 and 2, for each subgroup of participants used for analyses. As shown, participants were aged on average 29 years old (range: 18–65), with 54% of female. They were mainly employees or students and lived in cities or suburbs. Regarding their OCD symptomatology, the mean(SD) OCI-r score was 36.37 (12.82), ranging from 6 to 62. Most of the participants declared undergoing either no therapy (58%) or cognitive-behavioral therapy (CBT, 20%); and used either no treatment (52%) or antidepressant medication (30%). The intake frequency of treatments is shown in Table 2.

**Table 1:** Demographic information.

	OCD participants (n = 174)	OCD participants declaring a change (n = 129)	LSD and Psilocybin users (n = 90)
	Mean (SD) or %		
Age in years	29.49 (8.58)	29.40 (8.65)	29.31 (8.66)
*Gender*			
Female	54	50	44
Male	46	50	56
Height in cm	170.51 (10.74)	170.83 (11.32)	172.09 (11.37)
Weight in kg	69.98 (17.15)	70.85 (18.11)	73.95 (19.26)
*Survey language*			
English	76	85	84
French	24	15	16
*Social status*			
Employee	45	44	48
Student	23	24	25
Self-employed	16	15	13
Unemployed	10	12	10
Other	5	4	3
Retired	< 1	< 1	1
*Residence*			
City	61	61	59
Suburb	25	28	30
Country	14	11	11

**Table 2:** Health information.

	OCD participants (n = 174)	OCD participants declaring a change (n = 129)	LSD and Psilocybin users (n = 90)
	Mean (SD) or %		
OCI-r	36.37 (12.82)	36.94 (12.69)	36.30 (13.31)
OCD diagnosed by a health professional	62	67	70
Therapy *			
No	58	55	60
CBT	20	21	20
Other / No answer	10	12	11
Psychoanalytic therapy	5	5	3
Alternative therapies	3	4	3
Relaxation	1	0	0
Systemic therapy	1	2	1
EMDR	< 1	< 1	1
MBCT	< 1	< 1	1
Medications * and intake frequency			
No medication	52	52	53
Antidepressant	30	27	27
Every day	98	97	100
Several times a week	2	3	0
Anxiolytic	12	13	11
Every day	53	54	100
Several times a week	20	23	0
> once a month	27	23	0
Neuroleptic	3	< 1	0
Every day	100	100	0
Alternative medicines	8	9	10
*Comorbidities*			
Psychological troubles*	59	60	62
Anxiety disorders	48	48	54
Depressive disorders	36	36	42
Others	53	55	53
Neurological troubles	7	5	5
Physical troubles	29	29	31
Inflammatory diseases	10	12	13

*Multiple possible answers.

OCI-r = Obsessive compulsive inventory-revised version, CBT = cognitive behavioral therapy, EMDR: eye movement desensitization and reprocessing, MBCT: mindfulness-based cognitive therapy.

### Use of psychoactive substances in our population

To evaluate the use of psychoactive drugs in our population, a list was presented among which participants had to select the ones they had already used. As shown in Table 3, the most used substance categories among our OCD population were classic psychedelics (84%), entactogens (72%) and ketamine (49%); whereas NPS, delirogens and ibogaine were used by 22%, 16% and 2% of the participants, respectively. When looking at combinations of substances, we observed that 82% of classic psychedelics users had also used at least one other substance category. This proportion was 92% for entactogens users, 95% for ketamine users, 97% for NPS users and 100% for delirogens and ibogaine users. Among classic psychedelics users, 92% of participants used psilocybin mushrooms, 77% used LSD, 29% used DMT, 11% used mescaline and 9% used ayahuasca. Looking at combinations, we observed that 79% of psilocybin users had also used at least one other substance and this proportion was 94% for LSD users and 100% for DMT, mescaline or ayahuasca users.

**Table 3:** Use of psychoactive drugs in our OCD population.

Substances*	%	(n)
Classic psychedelics	84**	(146)
Psilocybin mushrooms	92 +	(135)
LSD	77 +	(113)
DMT	29 +	(42)
Mescaline	11 +	(16)
Ayahuasca	9 +	(13)
Entactogens	72**	(126)
MDMA	93 + +	(117)
Ecstasy	75 + +	(94)
Ketamine	49**	(86)
NPS	22**	(39)
Delirogens	16**	(28)
Salvia divinorum	100^O^	(30)
Ibogaine	2**	(4)

*Multiple answers possible.

**Percentage of participants with OCD (n = 174).

+ Percentage of classic psychedelics users.

+  + Percentage of entactogens users.

^o^Percentage of delirogens users.

LSD = Lysergic Acid Diethylamide, DMT = N,N-Dimethyltryptamine, MDMA = 3,4-Methylenedioxymethamphetamine.

### Changes in OCD symptoms induced by psychoactive drugs

To evaluate the impact of psychoactive drugs on OCD, participants were first asked whether they had experienced a change (irrespective of the valence of the change) in their OCD symptoms after taking one of the above-mentioned substances. Participants answering *Yes* had to select the substance which induced the strongest change in OCD symptoms (only one possible answer) and characterize the changes.

**Figure 2:**
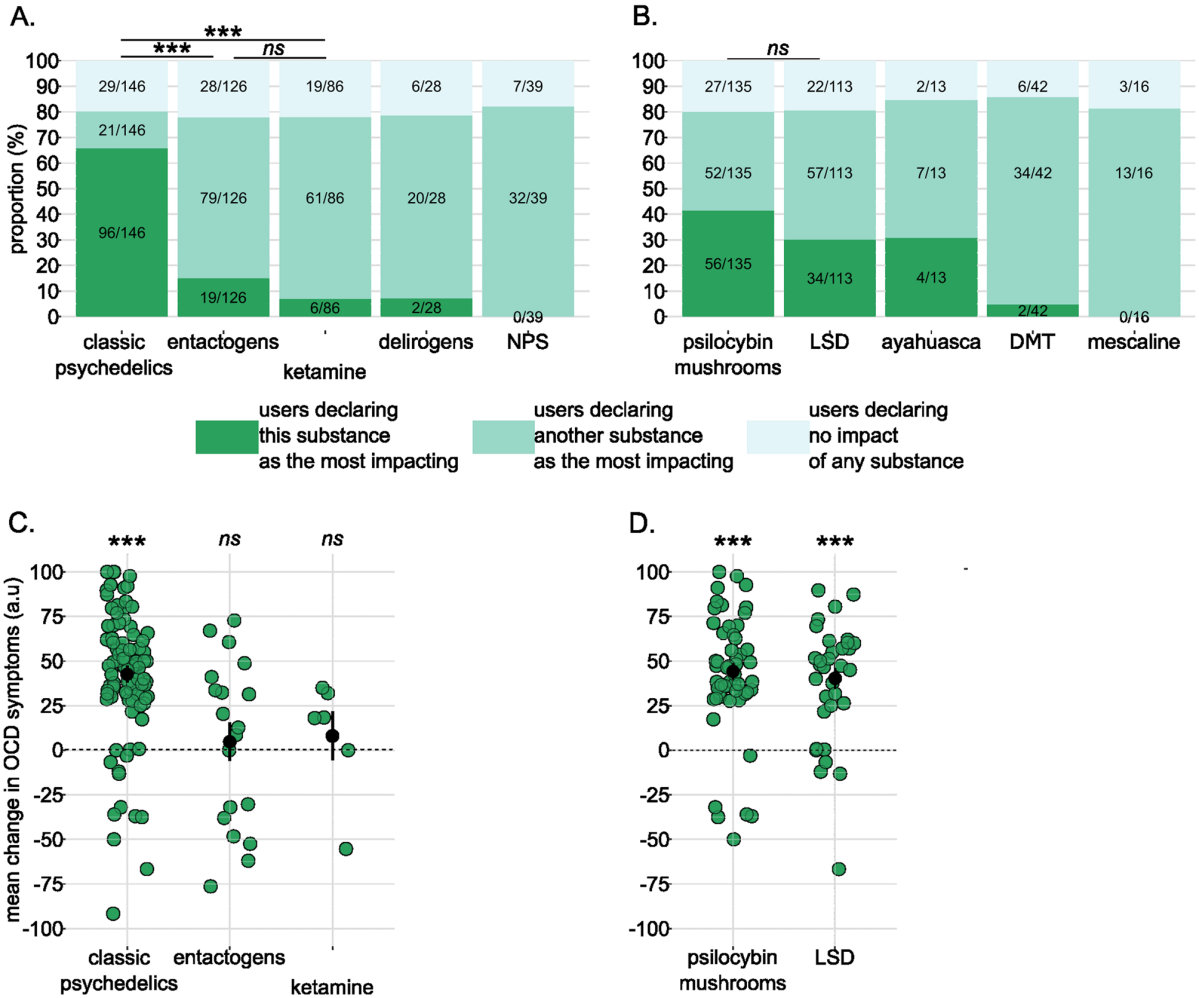
Changes in OCD symptoms induced by psychoactive drugs. (**A**) Proportion of users declaring the given substance (dark green), another substance (light green) or no substance (light blue) as the most impacting on OCD symptoms (irrespective of the valence of the changes). Proportions were compared using a χ^2^ test (**B**). Same representation for substances belonging to the classic psychedelics category. (**C**) Magnitude of changes in OCD symptoms for each substance category, expressed in arbitrary unit. Responses were provided on a scale ranging from -100 (= *worsening*) to + 100 (= *improvement*). Green dots represent the magnitude of the change per participant, black dots and bars represent the means and standard errors across participant, respectively. Significance was assessed using one-sample t-tests. (**D**) Same representation for psilocybin mushrooms and LSD users. The magnitude of the changes for NPS, delirogens, ayahuasca, DMT and mescaline were not computed due to small sample size (n < 5). ***P < .001, **P < .01, * P < .05, *ns* non-significant.

Among the participants who had used a given substance category, we calculated the proportion of participants declaring: the strongest change with this substance category, the strongest change with another substance category or no change with any type of substance. Results are reported in Fig. 2. Classic psychedelics were selected as the most impacting substances by 66% of the participants who declared using them. This proportion was 15% for entactogens, 7% for ketamine, 7% for delirogens and 0% for NPS. A χ^2^ test applied to categories with sufficient number of observations (n > 5) revealed that the proportion of participants declaring a substance category as the most impacting one varied according to the substance category (χ^2^(2) = 113.5, P < 0.001; Fig. 2A), with a significant difference in proportion between classic psychedelics and entactogens (P < 0.001) and between classic psychedelics and ketamine (P < 0.001) but no difference between entactogens and ketamine (P = 0.11). The same analysis was performed among classic psychedelics (Fig. 2B). Within this category, the proportion of users declaring the substance as the most impacting was 42% for psilocybin mushrooms, 30% for LSD, 31% for ayahuasca, 5% for DMT and 0% for mescaline. These proportions did not differ between psilocybin mushrooms and LSD (χ^2^(1) = 2.96, P = 0.08; other categories not tested due to insufficient number of observations).

When looking at the changes in OCD symptoms induced by the intake of each substance, we observed that mean changes were positive and significant for classic psychedelics (mean(SE) =  + 42.62 (3.79), t(95) = 11.24, P < 0.001) but not for entactogens (mean(SE) =  + 4.73 (10.69), t(18) = 0.44, P = 0.66) or ketamine (mean(SE) =  + 8.00 (13.65), t(5) = 0.59, P = 0.58; Fig. 1C). Within the classic psychedelics category, mean changes were positive and significant for both psilocybin mushrooms (mean(SE) =  + 44.22 (4.66), t(55) = 9.49, P < 0.001) and LSD (mean(SE) =  + 40.23 (5.95), t(33) = 6.76, P < 0.001; Fig. 1D).

Classic psychedelics appeared as the only substance category inducing a significant improvement in OCD symptoms, both in terms of the proportion of users declaring a change and the direction of the change. Therefore, we selected psilocybin mushrooms and LSD users (i.e., classic psychedelics users with a sufficient number of users) as our population of interest for the rest of the analysis (n = 90, characteristics in Table 1).

### Characteristics of the psychedelic experience

Data are shown in Table 4. For the mindset, 36% of the participants expected an improvement in their OCD symptoms. On the setting, a majority of participants declared that the intake occurred in *no particular context* (66%)*.* For the delay of onset and duration of the psychedelic experience, most of the participants reported a delay of onset *between 30 to 60 min* (62%) and a duration of *5–12 h* (48%).

**Table 4:** Characteristics of the psychedelic experience.

	%		Mean (SD)
Expectations*		**Acute general effects**	
Spiritual or religious purpose	73	Altered body sensations	80.61(16.38)
Personal development	69	Changes in vision or hearing	79.98(18.21)
Curiosity/Discovery	58	Euphoria	79.05(18.94)
Recreational experience	56	Emotional arousal	77.87(22.00)
OCD improvement	36	Insights	77.45(26.13)
None	1	Musical ecstasy	74.32(24.30)
Other expectations	1	Spiritual experience	74.30(29.89)
		Space time distortion	71.65(25.81)
		Trauma resolution	62.67(30.53)
**Context of the dosing**		Hallucination	58.11(31.76)
No particular event	66	Bodily boundaries dissolution	50.97(30.01)
Recreational setting	16	Ego dissolution	47.95(32.68)
Ceremonial setting	18	Confusion	35.39(30.40)
With therapeutic support	50	**Acute unpleasant effects**	
Retreat	29	Difficulty concentrating	44.85(30.12)
Shamanic type ceremony	21	Nausea	38.00(31.29)
Clinical research	0	Feeling of heat	35.99(31.76)
		Anxiety	34.14(30.38)
		Discomfort	34.09(29.75)
**Delay of onset of acute effects**		Feeling of coldness	33.54(33.55)
Less than 30 min	17	Depersonalization	31.64(31.98)
0 to 15 min	7	Memory impairment	31.39(30.04)
15 to 30 min	93	Revival of traumatic memories	31.16(32.94)
30 to 60 min	62	Sadness	28.14(30.93)
More than 60 min	17	Indigestion	26.02(29.39)
No answer	4	Sedation	23.71(27.16)
		Shaking	22.33(26.65)
		Panic	21.19(30.74)
**Duration of acute effects**		Tachycardia	17.41(23.34)
Less than 5 h	28	Headache	15.25(23.20)
1–3 h	20	Diarrhea	14.98(23.24)
3–5 h	80	Vomiting	12.13(21.51)
5–12 h	48	Constipation	9.79(18.47)
> 12h	20	Self-aggressive behavior	8.11(19.50)
I don't know	4	Aggressive behavior	4.41(13.68)

*Multiple possible answers.

**Pleasantness score = 100 – mean(Acute unpleasant effects).

For general effects, mean ratings across-participants were all above 50 (except for *confusion* and *ego dissolution*) whereas for unpleasant effects, mean ratings across-participants were all below 50. The resulting intensity scores reached a mean(SE) of 67.07 (1.61) across participants, ranging from 20.77 to 100; and the pleasantness scores reached a mean(SE) of 73.91 (1.92), ranging from 21.1 to 100.

### Proxy for the dose

**Figure 3:**
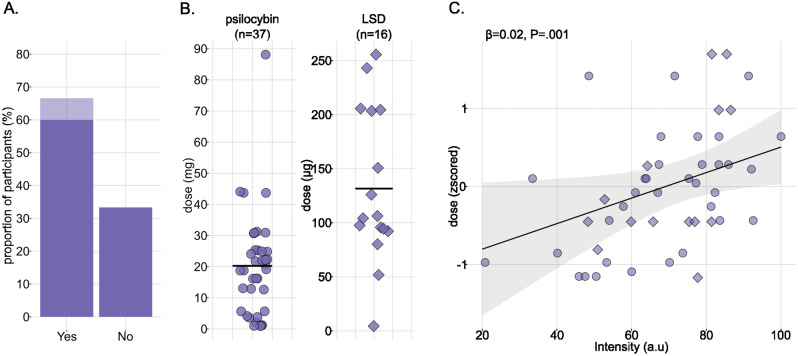
Relating self-reported dose to intensity of acute effects. (**A**). Percentage of participants who declared knowing the substance dose (n = 60) or not (n = 30). Light purple data represent participants discarded because of uninterpretable dose (n = 7). (**B**). Distribution of doses for psilocybin (left panel) and LSD (right panel). Black bars represent means. (**C**). Dose of substance as a function of the intensity of the acute effects. Doses are expressed as z-scores to include both psilocybin mushrooms and LSD in the same analysis. One dot is one participant, the line and ribbon represent the estimated regression line and its 95% confidence interval. Note that β represents the regressor for intensity in the statistical model used to predict the dose.

When asked if they knew the substance dose they had taken, 67% of the participants answered positively (Fig. 3A). Based on the provided dosage and associated unit, 8% of them were discarded due to missing or absurd information (e.g., participants declaring the intake of 4g of LSD). For psilocybin mushrooms, most of the doses were expressed as grams of dried mushrooms, which we converted into milligrams of psilocybin by multiplying by a 0.0063 factor (appropriate for *Psilocybe cubensis* type of mushrooms according to^30^). Following this transformation, the mean(SD) dose of psilocybin was 20.30 (16.74)mg, ranging from 1 to 88 and the mean(SD) dose of LSD was 131.56 (69.85)μg, ranging from 10 to 250 (Fig. 3B).

Since doses were exploitable in a subset of participants only, we looked for a variable available in all participants that could constitute a proxy for the dose, by running a linear regression with 3 factors (delay of onset, duration, intensity of acute effects; all GVIF < 2). We did not include the pleasantness of acute effects as a regressor since previous study did not observe its association with the dose^31^. Five participants were removed from the analysis because of missing data (n = 4 for the delay of onset and n = 1 for the duration), leading to 48 participants included in the analysis. Results showed that the intensity of acute effects was positively associated with the dose (β = 0.02, t = 3.48, P = 0.001; Fig. 3C). None of the other regressors significantly predicted the dose (Delay of onset: β_less30-30to60_ = 0.09, t = 0.34, P = 0.73, β_more60-30to60_ = 0.06, t = 0.22, P = 0.82; Duration: β_5to12h-more12h_ = 0.37, t = 1.28, P = 0.21, β_less5h-more12h_ = 0.26, t = 0.82, P = 0.41). This analysis shows that in our dataset, the intensity of acute effects can be used as a proxy for the dose, with increasing doses leading to more intense experiences.

### Predicting the magnitude of OCD improvement

**Figure 4:**
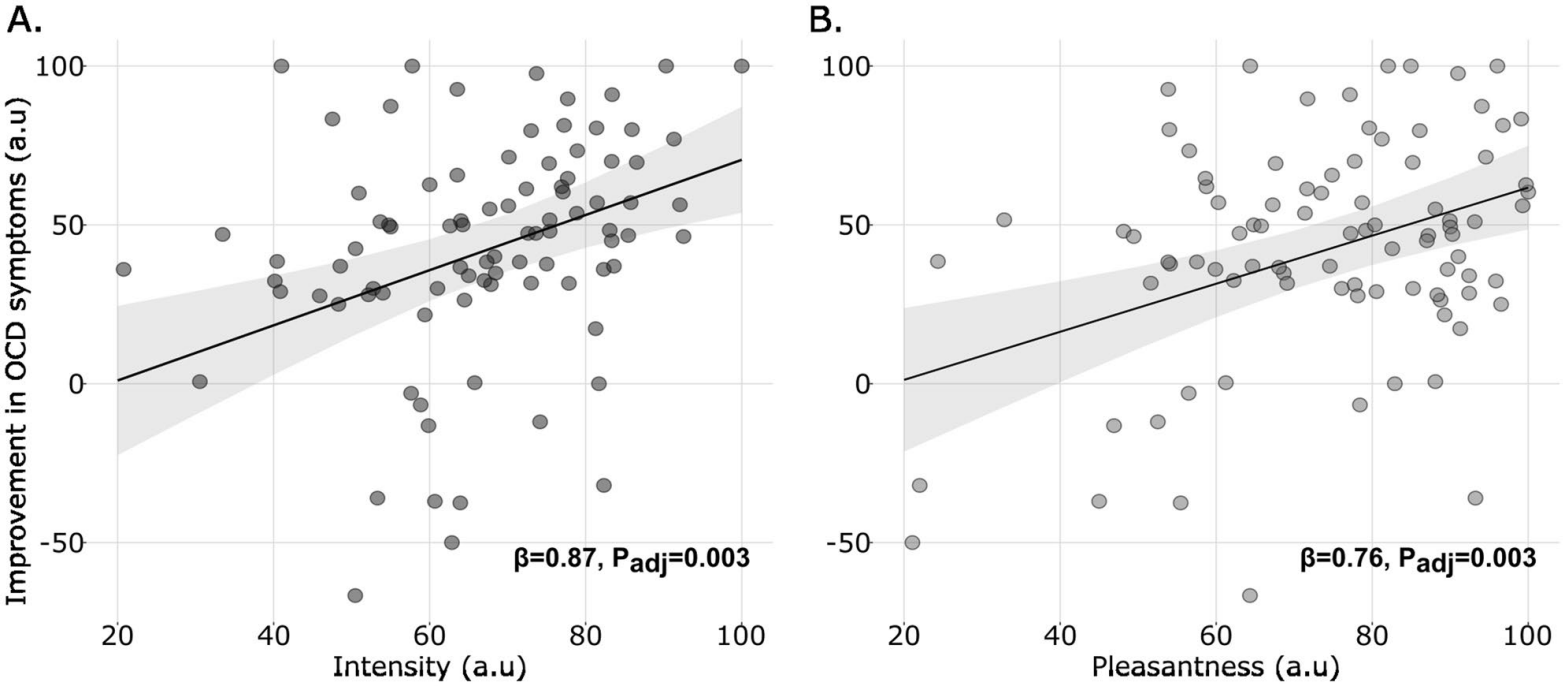
Modeling the magnitude of OCD improvement. OCD improvement as a function of intensity (A) and pleasantness (B) of acute effects of psychedelics, assessed in 90 participants. One dot is one participant, the line and ribbon represent the estimated regression line and 95% confidence intervals. Note that β represents the regressor for intensity (respectively pleasantness) in the linear regression used to predict the magnitude of OCD improvement.

To study which factors predicted the magnitude of OCD improvement, a linear regression including 6 independent variables (substance, mindset, context, intensity and pleasantness of acute effects, OCI-r score; all GVIF < 2) was used. One participant was removed from the analysis because of missing data, ending in 89 observations included. In our dataset, the magnitude of OCD improvement was positively associated with both the intensity (β  = 0.87, t = 3.86, P_adj_ = 0.003; Fig. 4A) and pleasantness (β  = 0.76, t = 3.96, P_adj_ = 0.003; Fig. 4B) of acute effects. None of the other factors was significant (substance: β_Psilo-LSD_ = 5.67, t = 0.77, P_adj_ = 0.67; mindset: β_Expectation-NoExpectation_ = 2.80, t = 0.36, P_adj_ = 0.81; setting: β_Recreational-Noparticular_ = -9.09, t = -0.9, P_adj_ = 0.61; β_Ceremonial-NoParticular_ = 6.27, t = 0.69, P_adj_ = 0.70; OCI-r score: β = 0.03, t = 0.1, P_adj_ = 0.92). This analysis shows that the improvement in OCD symptoms was not associated with the mindset or context of intake but was larger for more intense and more pleasant acute effects.

### Pleasantness of acute effects

Since the pleasantness of acute effects appeared as a predictive factor of OCD improvement magnitude, a linear regression with 5 regressors (substance, mindset, context, intensity of acute effects, OCI-r score; all VIF values < 2) was used to study which factors were predictive of pleasantness. One participant was discarded from the analysis because of missing data, ending in 89 observations included in the analysis. None of the factors included in the analysis significantly predicted the pleasantness of acute effects (substance: β_Psilo-LSD_ = -3.75, t = -0.89, P_adj_ = 0.61; mindset: β_Expectation-NoExpectation_ = 5.76, t = 1.30, P_adj_ = 0.54; setting: β_Recreational-NoParticular_ = -3.82, t = -0.66, P_adj_ = 0.70; β_Ceremonial-NoParticular_ = 1.27, t = 0.24, P_adj_ = 0.84; intensity: β = -0.18, t = -1.37, P_adj_ = 0.54; OCI-r score: β = -0.29, t = -1.91, P_adj_ = 0.39).

### Persistence of the improvement in OCD symptoms

**Figure 5:**
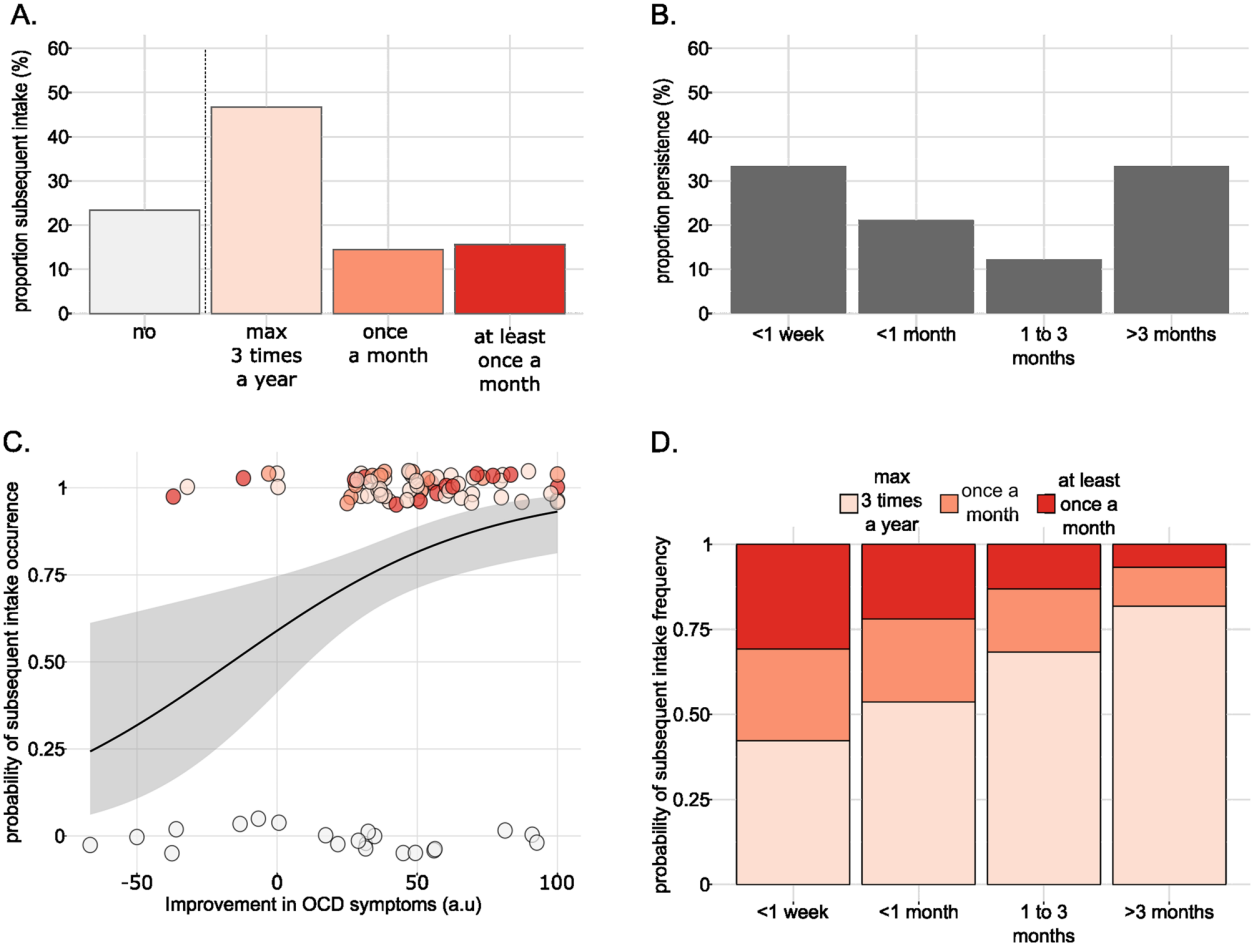
Modeling subsequent intake probability and frequency**.** (**A**) Repeated use of the substance. (**B**) Persistence of substance-induced OCD improvement. (**C**) Estimated probability of subsequent intake as a function of OCD improvement magnitude, as modeled using a logistic regression (n = 90). One dot is one participant; the line and ribbon represent the regression curve and associated standard error. **D**. Estimated distribution of subsequent intake frequency according to the persistence of OCD improvement, as modeled using an ordinal regression (n = 69).

Improvement in OCD symptoms was mostly reported to persist for *less than a week* and *more than 3 months*, with 33% (n = 30) in each. An additional 21% of participants (n = 19) selected *between 1 week and 1 month* and the remaining 12% (n = 11) selected *between 1 and 3 months* (Fig. 5B). We acknowledge that reports on persistence are not precise enough since we do not know when the intake occurred relative to the filling of the survey. Notably, participants who filled the survey one week after the intake were not able to evaluate the persistence at three months. But given the importance of this parameter, we still searched for determinants. An ordinal regression with 5 independent variables (substance, intensity and pleasantness of acute effects, magnitude of OCD improvement, OCI-r score; all GVIF < 1.5) was used, including 90 observations. Results showed that none of the regressors significantly predicted the persistence of the improvement (substance: β_Psilo-LSD_ = -0.44, z = -1.06, P_adj_ = 0.54; intensity: β = 0.02, z = 1.20, P_adj_ = 0.54; pleasantness: β = -0.02, z = -1.52, P_adj_ = 0.54; magnitude of OCD improvement: β = 0.003, z = 0.43, P_adj_ = 0.78; OCI-r score: β = -0.02, z = -1.19, P_adj_ = 0.54).

### Subsequent intakes and their frequency

When participants were asked whether they had taken the substance again, 23% (n = 21) answered negatively. Among the 77% who took it again, 47% (n = 42) reported an intake frequency of *at most 3 times a year*, 14% (n = 13) reported *once a month* and 16% (n = 14) *at least once a week* (Fig. 5A). We first searched for factors predicting the occurrence of subsequent intakes (irrespective of the frequency), using logistic regression with 6 independent variables (substance, intensity and pleasantness of acute effects, magnitude and persistence of OCD improvement, OCI-r score; all GVIF < 2) on 90 observations. The only significant factor associated with the occurrence of subsequent intake was the magnitude of OCD improvement (β = 0.03, z = 2.92, P_adj_ = 0.03; Fig. 5C) whereas none of the other factors was significant (substance: β_Psilo-LSD_ = -0.30, t = -0.49, P_adj_ = 0.77; intensity: β = -0.02, z = -1.08, P_adj_ = 0.54; pleasantness: β = -0.02, z = -1.09, P_adj_ = 0.54; persistence: β_1weekto1month-less1week_ = -0.46, z = -0.59, P_adj_ = 0.72; β_1to3months-less1week-_ = -1.45, z = -1.43, P_adj_ = 0.54; β_more3months-less1week_ = -0.23, z = -0.32, P_adj_ = 0.81; OCI-r score: β = 0.03, z = 1.30, P_adj_ = 0.54). This result shows that the probability of subsequent intake increases with stronger improvement in OCD symptoms.

In the participants who took the substance again (n = 69), we then modeled the frequency of intake using an ordinal regression including 5 regressors (substance, intensity and pleasantness of acute effects, magnitude and persistence of OCD improvement; all GVIF < 2). We observed that subsequent intake frequency was significantly associated with the persistence of OCD improvement, with improvements persisting *more than 3 month*s increasing the probability of being in the category *at most 3 times a year* (β_more3months-less1week_ = -1.76, z = -2.53, P = 0.01; Fig. 5D). None of the other factors was significant (substance: β_Psilo-LSD_ = 0.30, z = 0.55, P = 0.58; intensity: β = -0.001, z = -0.08, P = 0.94; pleasantness: β = 0.02, z = 1.39, P = 0.17; magnitude of OCD improvement: β = -0.007, z = -0.65, P = 0.51).

Finally, we explored the substance dose used by participants who declared taking the substance *at least once a week*. Among them, 57% (n = 8) used microdosing with doses lower than 10 µg for LSD and 4 mg for psilocybin, 21% (n = 3) did not provide any information and the remaining 21% (n = 3) reported using regular doses (250 µg for LSD and above 35 mg for psilocybin).

This last analysis shows that in our dataset, the occurrence and frequency of subsequent intakes were not related to the intensity or pleasantness of acute effects but to the improvement in OCD symptoms and its persistence.

## Discussion

In this survey, we assessed, in a sample of participants suffering from OCD symptoms, the subjective efficiency of different psychoactive drugs in reducing symptoms. When considering the different substance categories, we observed that the proportion of users reporting a change in OCD symptoms (irrespective of its valence) was higher with classic psychedelics than with any other substance category. And when characterizing these changes, we observed that only classic psychedelics were associated with significant improvements in OCD symptoms. Consequently, we focused on classic psychedelics users for which we had enough participants, meaning psilocybin mushrooms and LSD users, to study the determinants of the therapeutic effect. We observed that its magnitude was associated with the intensity and pleasantness of acute effects, with the intensity being correlated with the substance dose. Regarding the persistence of the therapeutic effect, over 30% of participants reported effects lasting for more than three months. However, no significant predictor was detected that could explain the variability of reports. Finally, substance usage appeared to be tuned to the therapeutic effect. Indeed, the occurrence of subsequent intakes was predicted by the magnitude of OCD symptoms improvement, with participants with stronger therapeutic effect being more likely to take the substance again. And among the participants who used the substance again, the frequency of subsequent intakes was related to the persistence of the therapeutic effect, with long-lasting (> 3 months) improvements predicting a lower frequency of intake.

As shown, a higher proportion of participants chose classic psychedelics as the most efficient for OCD symptoms when compared to other substances. The self-assessment of the therapeutic effect makes it prone to subjectivity bias, but a vast majority of classic psychedelics users, in our sample, *perceived* an improvement in their medical conditions following the intake of the substance. The fact that many participants used multiple substances brings additional credits to their choice, since they were likely able to compare between substances. Interestingly, ketamine, a dissociative substance with anesthetic properties at high doses, did not show up as an effective substance in alleviating OCD symptoms. Previous results on the use of ketamine in OCD are mixed^32^, but our results are difficult to compare since recreational use differs from therapeutic guidelines in terms of enantiomer, dose and mode of administration. Similarly, DMT and mescaline were not selected as effective for OCD symptoms. Despite being categorized as serotoninergic hallucinogens, DMT and mescaline have phenomenological and pharmacological profiles that seem to differ from LSD or psilocybin^33^. Among other differences, DMT has no affinity for the serotoninergic receptor 2C, as well as a lower hallucinogenic potency and duration of action in human^34^. Mescaline on the other side, seems to have combined psychedelic and entactogenic properties^35^ and does not act on raphe neurons^36^. Finally, within the classic psychedelics category, we did not observe any difference between LSD and psilocybin, which share multiple chemical properties but also differences such as their affinity for dopaminergic receptors^37^.

When modeling the magnitude of the therapeutic effect, we observed that it was predicted by the intensity of acute effects. The evaluation of acute effects is intrinsically subjective, and the retrospective design of the study could have affected the scorings due to recall bias. However, the observation of an already described and therefore expected link between the intensity of acute effects and the dose^38^, suggests that their evaluations were somewhat trustful. This observation further suggests that moderate to high doses are necessary to obtain a therapeutic effect, which is coherent with preliminary data in OCD^17^, as well as in other pathologies^39–41^. The intensity of the subjective experience is not, however, solely determined by the dose, but also by its quality (i.e., content of the subjective experience). And how the content of the subjective experience affects the therapeutic effect remains an open question in the field, which calls for a transdisciplinary approach combining phenomenology and ethnography. We should mention that 10% of participants reported a worsening of OCD symptoms. Whether similar proportion are observed in well-controlled clinical trials is an important matter, which would call for an evaluation of risk factors.

In our survey, the magnitude of the therapeutic effect was also correlated with the pleasantness of acute effects, but we suspect a carry-over effect. Indeed, the scoring of the therapeutic effect might have been strongly influenced by the global impression of the participants on the acute subjective experience, regardless of any causal relation. Additionally, previous survey has reported, in healthy subjects, the lack of relationship between the pleasantness of acute effects and the enduring well-being induced by psilocybin^23^. However, this association should be investigated further in patients since one of the most prevalent adverse effects reported during the acute experience is anxiety^42^, a symptom for which OCD patients might be particularly at risk. Investigating the factors influencing the pleasantness of the subjective experience is an important matter, not only because it might affect therapeutic effects, but also because it has repercussions on the feasibility of administrating psychedelics in OCD patients. We also included as factors in the model the mindset and context of the intake and did not observe any impact of it. However, we acknowledge that the assessment of these parameters was rather coarse in our study, and it seems reasonable to think that the context of the intake at least, might impact the pleasantness of the experience. Finally, since LSD and psilocybin have been shown to be efficient in reducing depressive symptoms, which were also reported by 42% of participants in our sample, it would be interesting in future trials to specifically assess the effect of classic psychedelics on different dimensions including obsessions, compulsions and depression.

In addition to the magnitude of the therapeutic effect, another major component in assessing the efficacy of a treatment is the persistence of the effect. Strikingly, we had 30% of participants reporting therapeutic effects lasting more than three months. There is, to our knowledge, no reported data on the persistence of the psychedelic-induced therapeutic effects in OCD apart from case studies^7,10,11,43,44^. But this is coherent with studies in other pathologies such as depression showing effects persisting from multiple weeks to several months^41,45,46^. As mentioned in the results section, the persistence data were imprecise since we did not have access to the time delay between the intake of the psychoactive drugs and the filling of the survey. This might have impacted the proportion of participants declaring short-lasting effects, with people filling out the survey shortly after the intake of the substance not being able to assess the maintenance of the therapeutic effect in the long term. The mechanism through which psychedelics may induce long-term therapeutic effects also remains unknown. One candidate is the neuroplastic property of psychedelics, with increased neuronal growth and synaptogenesis observed both in vivo in rodents and in vitro in different species^47–49^. In rodent, these structural modifications lasted up to a month, but were not specific to classic psychedelics since it was observed with multiple components including ketamine^47^.

The last factor that we examined was the occurrence and frequency of subsequent intakes. We did not observe any apparent case of substance abuse from participants’ reports, with no or very moderate intake repetition in most of them. This is coherent with the literature reporting no reinforcing properties of classic psychedelics, neither in humans nor in animals^37^. Importantly, in our dataset, the occurrence of subsequent intakes and their frequency seemed to be closely related to the magnitude and persistence of the therapeutic effect. The causal relationship between the occurrence of subsequent intake and the therapeutic effect cannot be inferred, mainly due to the retrospective nature of our study. However, it was striking to see no apparent relationship between the pleasantness of the acute effects and the frequency of subsequent intake.

Some methodological limitations must be considered beforehand. In general, web-based surveys are subject to bias in participant selection. In the context of our study, participants were likely to be favorable to the use of psychedelic drugs, which might have had an impact on their trustfulness and might have induced proselytism when completing the survey. Participants with a pleasant acute experience and positive outcome (i.e., improvement in OCD symptoms) were also more likely to enter the survey, limiting the generalizability of our results. Still, we attempted to objectively evaluate symptom severity using the OCI-r. The answers provided by our participants with OCD symptoms were also coherent with the literature on OCD management, with the most used therapy and medications in our sample being cognitive-behavioral therapy and antidepressant, which are the two recommended first-line therapeutic approaches for OCD^3^. Also, the ratio between male and female as well as the rate of comorbidities were very similar in our sample when compared to epidemiological studies on OCD^50,51^.

In conclusion, our dataset suggests that classic psychedelics are potentially efficient in reducing OCD symptoms, with an impact of the dose on the magnitude of the therapeutic effect, and little risk of substance abuse. Our results also highlight the need to carefully assess the persistence of the therapeutic effect in OCD. All limitations related to online surveys should be kept in mind, and these results should not be generalized to the entire OCD population until the outcome of randomized controlled clinical trials confirms our observations.

## Data Availability

The full version of the survey is available at the following link: https://osf.io/x2sk7/?view_only=5ef581a60b7a4d5685a2c233a0435a15.
